# Dental Economics

**Published:** 1912-02-15

**Authors:** N. S. Coyne


### DENTAL ECONOMICS.

BY N. S. COYNE, D.D.S.

Much is being written and much is being said in this present age in regard to the raising of the status of Dentistry. I wonder how many practising dentists ever asked themselves why dentistry as a profession had not, in years gone by, been looked on as the noble profession that it is or shall be. To my mind there are two main reasons why Dentistry has not been held in as high esteem by the public as its members have desired. They are these: incompetent service, and Jack-knife-carpenter's fees.

Many men will no doubt resent the first reason I have advanced, but that will not change my opinion in the least. In spite of any amount of protests the fact remains that the average dentist does not give *nearly* as good service as the public are in need of, as it is possible to give, or as is expected of dentists. Not that the Academic training is at fault, not that our course is too short; but rather because we do not give the best, or nearly the best, services that this skill and ability will admit. I don't mean the country dentist alone—not by any means—but the average dentist in city or town.

The question that now arises naturally is, Why is such the case? The answer is simple. The average dentist can't afford to expend the time to execute his operations properly, or nearly so, at the fee "generally charged" in his community—rural or urbane. But some men argue this way, "A filling is a filling, they all look alike to the public; they don't appreciate an extra effort." Such an argument as this by no means justifies a man in rendering poor service. When a man obtains a license to practice dentistry he is supposed to give as good service as his skill and ability allows, not as good service as the public will appreciate. But, to go beyond this, the day for saying "A filling is a filling" is, or should be, past and gone. Fillings can no longer be doled out as boots or hats at so much per.

Again, a dentist never wants to run away with the idea that the patient doesn't see and realize when the operator is painstaking and thorough. Patients will, in fact, remark to the dentist, "My! you're awfully particular," or, "You're certainly thorough in your work," or, "You must have a lot of patience or you couldn't spend so much time on one tooth," or some similar remark.

But so many men say, "We could never save a cent if we were to spend so much time on detail." I'll admit we couldn't at 50 cents or 75 cents or $1 a filling. There's an old family saying, "A thing that's worth doing is worth doing well." This is particularly true of operations in the oral cavity. Worth doing well—from the dentist's viewpoint—and worth doing well, decidedly worth doing well, from the patient's viewpoint. If its worth doing well from the patient's viewpoint, its worth paying well for. It seems to me this is the only logical, practical conclusion to come to.

But many practitioners persist in saying, "It takes a long time to educate the public." Supposing it should—to my mind that's what we're here for. While we're educating the public we can be collecting a proper fee for the service we render—so that the dentist is benefited as well as the patient. My argument is simply this, "Be thorough, both in the execution of all operations, and in assessing the fee." Why should we be any more slipshod in the latter than in the former? If a patient presents, requiring the incisal corner of a central restored, be thorough—restore the corner to its proper anatomical shape, although it should take three of four times as long and "a bit more gold" than would be necessary in "dubbing off" the corner in order to be able to do it for $2. If it's necessary to charge $8 or $10 charge it. The patient has received much better value in this latter way than he would at $2 or $3, and the work only half done. If a case presents with a decayed molar, dentine deeply stained, even approaching the pulp so that protection is necessary; be thorough although it may be necessary to cut and chisel and excavate long and carefully; yes, even if it should be necessary to spend an hour or an hour and a half to prepare and fill with amalgam. Don't back up—prosecute your operation with the same thoroughness that you were compelled to in the college infirmary. There's no "short cut" that I know of—only one way; that's the right way, and, when done, don't get behind the patient and bite your nail and wonder if you dare charge more than 75 cents. Don't choke back a sigh and work your pulse up to 200 for fear your patient will leave for *good*—never come back—go to Dr-Do-it-Cheaper if you dare charge $1. Charge what its worth. If you don't know what its worth take half a day off and figure it out; and, by the way, if you have spent an hour and a half and charge less than $3, you're going to be a subject of Charity when you're sixty if you don't die of starvation long before.

But, again, another argument put forth is this: "It's all right for men in the city to do good work and charge for it, but people in the country aren't built that way—its just the 'city folk' that one can work that system on." And the dentist in the city says, "The other fellow round the corner extracts teeth and does 'plate work,' I've got to do it or I won't get the patronage. We've all got to do the same."

This kind of talk is all bosh. In the first place the country people are entitled to first-class dental service as well as city folk are, and country people generally want it. By this I mean thorough, skilful, honest work. Why should country people want their teeth "plugged" with "silver" while city people have their's properly treated and properly restored to their natural functional power? On the other hand, why should the city practitioner render incompetent, inferior service at a cheap fee simply because the fellow round the corner does the same. The patient has presented herself to *you*—not to the fellow around the corner. She evidently wants your service, and it's up to you to give that patient such service as will give her a desire to employ you in the future, and not look elsewhere for a dentist. The fee won't diive the patient to some other fellow if the services rendered have been honest and competent; but inferior service *will* drive the patient to some other dentist, no matter how low or beggarly your fee may have been. I believe the dentists' motto should always be: "Good Services and Good Fees."

A few weeks ago a deputation from the Dominion Canners waited on our Town Council in regard to a canning factory proposition for our town. In the course of the interview, I was struck with a remark from one of the deputation (the President of the company) as to their aim. He said: "Gentlemen, our aim is cleanliness and quality. We require an abundance of water for cleanliness, and we require your best natural products, in order that we may produce quality." Now, surely, if the aim of a company preparing food for the human race is cleanliness and quality, is it not as necessary that the aims of the dentist, who treats the organs that are given us to masticate that food with should be as high as that of the manufacturer? Is it not reasonable to say that the aim of every dentist should be "cleanliness" and "quality?"

But I would just like to mention that while the aim of the Dominion Canners is as I have stated, I note with equal interest that the same company operate their entire concern on a money-making basis. The Dominion Canners sell their goods for a good price, made possible by first producing the quality. This may seem to some like rather a crude comparison, and even unethical, but I believe if more dentists looked at the practice of dentistry through their glasses there would be fewer unethical dentists.—*Oral Health.*

